# Supplementary material for: Identifying STEDable BF2-Azadipyrromethene Fluorophores
Source: Molecules. 2023 Feb 2;28(3):1415. doi: 10.3390/molecules28031415 (PMC9919209; doi:10.3390/molecules28031415)
Supplement: Supplementary file 1 [file molecules-28-01415-s001.zip › molecules-2199681-supplementary.pdf]

Supplementary Information

Identifying STEDable BF_2-Azadipyrromethene Fluorophores

Niamh Curtin, Massimiliano Garre, Dan Wu, Donal F. O'Shea*

Department of Chemistry, RCSI, 123 St Stephen's Green, Dublin 2, Ireland.

Supplementary Information

Contents

**Figure S1**	Fluorescence decay curves of **1-4**.	**S3**
**Figure S2**	Excitation scans for emission peak 775 nm for **1-4**.	**S3**
**Figure S3**	Phasor plot of **1-4** in aqueous solutions.	**S4**
**Figure S4**	Normalised emission intensity induced by EL and DL for **2-4**.	**S4**
**Figure S5**	CLSM and STED imaging of LDs using **1**.	**S4**
**Figure S6**	Comparative CLSM and STED live imaging of **1**.	**S5**
**Figure S7**	Fluorophore **1** measurements of resolution improvement.	**S6**
**Figure S8**	CLSM and STED image with **1** used for Fig. 6.	**S7**
**Figure S9**	CLSM and STED imaging of **1** with increased spatial resolution analysis.	**S7**
**Figure S10**	STED z-stack image with **1**.	**S8**
**Figure S11**	Continuous live cell STED imaging with **1**.	**S9**
**Figure S12**	Comparative CLSM and STED imaging with **2**.	**S10**
**Figure S13**	Comparative CLSM and STED imaging with **3**.	**S11**
**Figure S14**	CLSM and STED imaging of **2** with increased spatial resolution analysis.	**S11**
**Figure S15**	Image resolution analysis for **1**.	**S12**
**Figure S16**	Image resolution analysis for **2**.	**S13**

**Figure S17**	Image resolution analysis for **3**.	**S14**
**Figure S18**	Live CLSM and STED image with **4**.	**S15**
**Figure S19**	Imaging with **4** using DL alone.	**S15**
**Figure S20**	Repeat experiments imaging CLSM and DL alone with **4**.	**S16**
**Figure S21**	Imaging with **4** by CLSM and DL alone with overlay.	**S16**

Movie Legends

**Movie S1A**	SI Fig. S11 continuous live cell STED imaging.
**Movie S1B**	SI Fig. S11 continuous live cell STED.
**Movie S2**	SI Fig. 10D cell z-stack movie.
**Movie S3**	Additional cell z-stack movie.
**Movie S4**	Fig. 10A cell z-stack movie.
**Movie S5A**	Fig. 10B (i-iii) z-stack movie of cell.
**Movie S5B**	Fig. 10B (i-iii) 3D movie of cell.
**Movie S6**	Fig. 10B (iv) z-stack movie of cell.
**Movie S7**	SI Fig. 10C z-stack movie of cell.
**Movie S8A**	Additional z-stack movie of cell.
**Movie S8B**	Additional 3D movie of cell.
**Movie S9**	SI Fig. S19 DL power movie of cell treated with **4**.

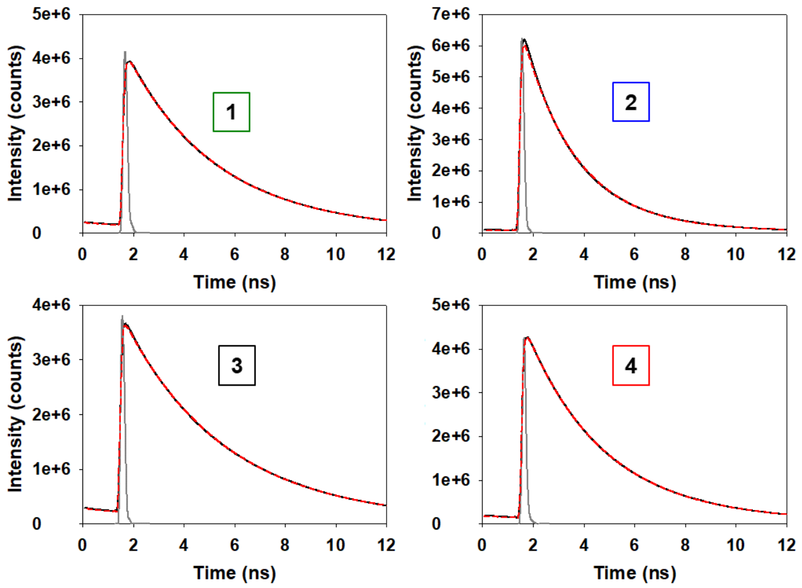

Figure S1. Fluorescence decay curves of fluorophores (5 μM) **1** (3.6 ns), **2** (2.2 ns), **3** (3.9 ns) and **4** (3.1 ns) in aqueous solution; counts (black line), instrument response function (grey line), line of best fit (red line).

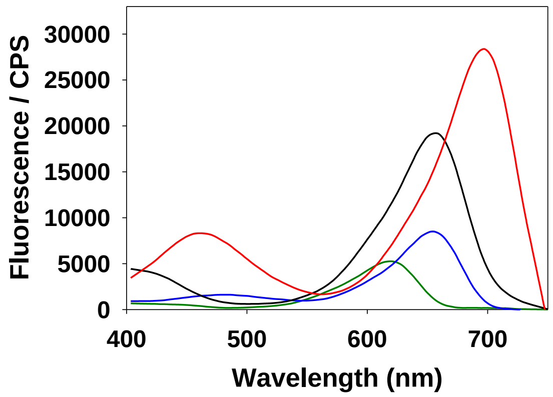

Figure S2. Excitation scan for 775 nm of fluorophores (5 μM) **1** (green), **2** (blue), **3** (black) and **4** (red) in aqueous solution, slit widths excitation 1 nm, emission 2 nm.

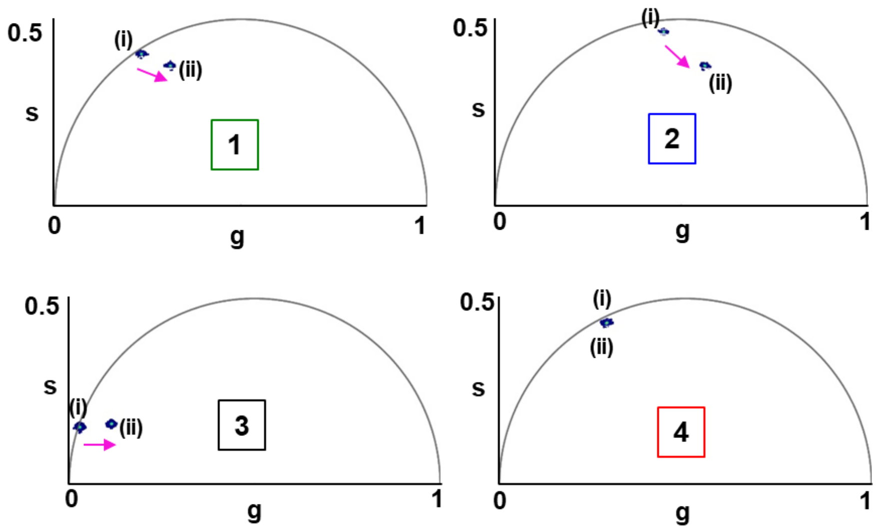

Figure S3. Changes in phasor plot of fluorophores (5 μM) **1-4** in aqueous solution when excited by (i) EL and (ii) EL + DL (30 %), pink arrows show shift in lifetime.

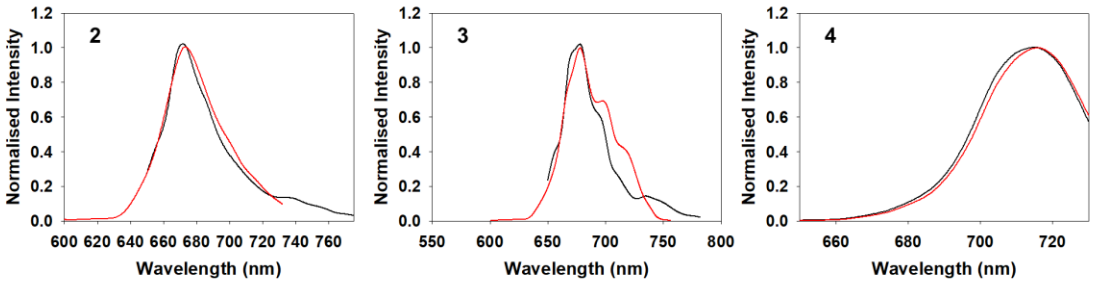

Figure S4. Normalised emission intensity caused by EL (black line) and DL (30%, red line) for fluorophores (5 μM) **2-4** in aqueous solution.

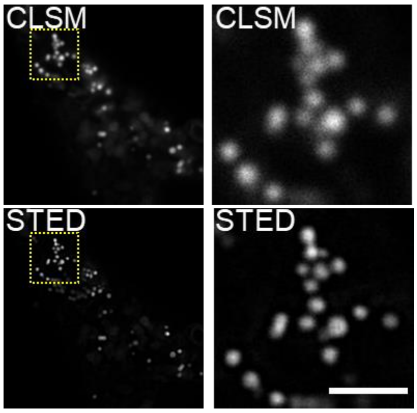

Figure S5. CLSM and STED imaging of MDA-MB 231 cell containing LDs incubated with **1** (5 μM) for 3h, yellow box expanded, scale bar 2 μm.

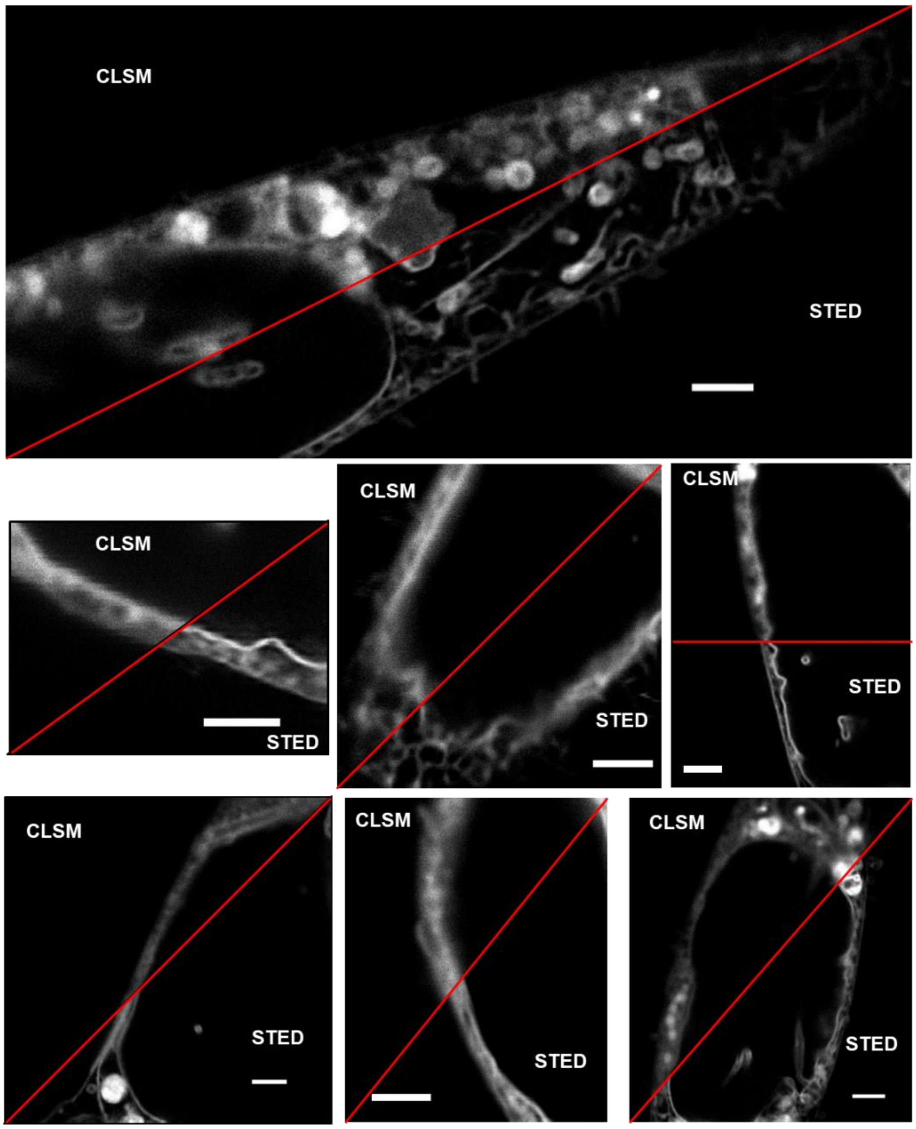

Figure S6. Comparative CLSM and STED imaging of **1** (5 µM) following 3 h incubation in different live MDA-MB 231 cells.

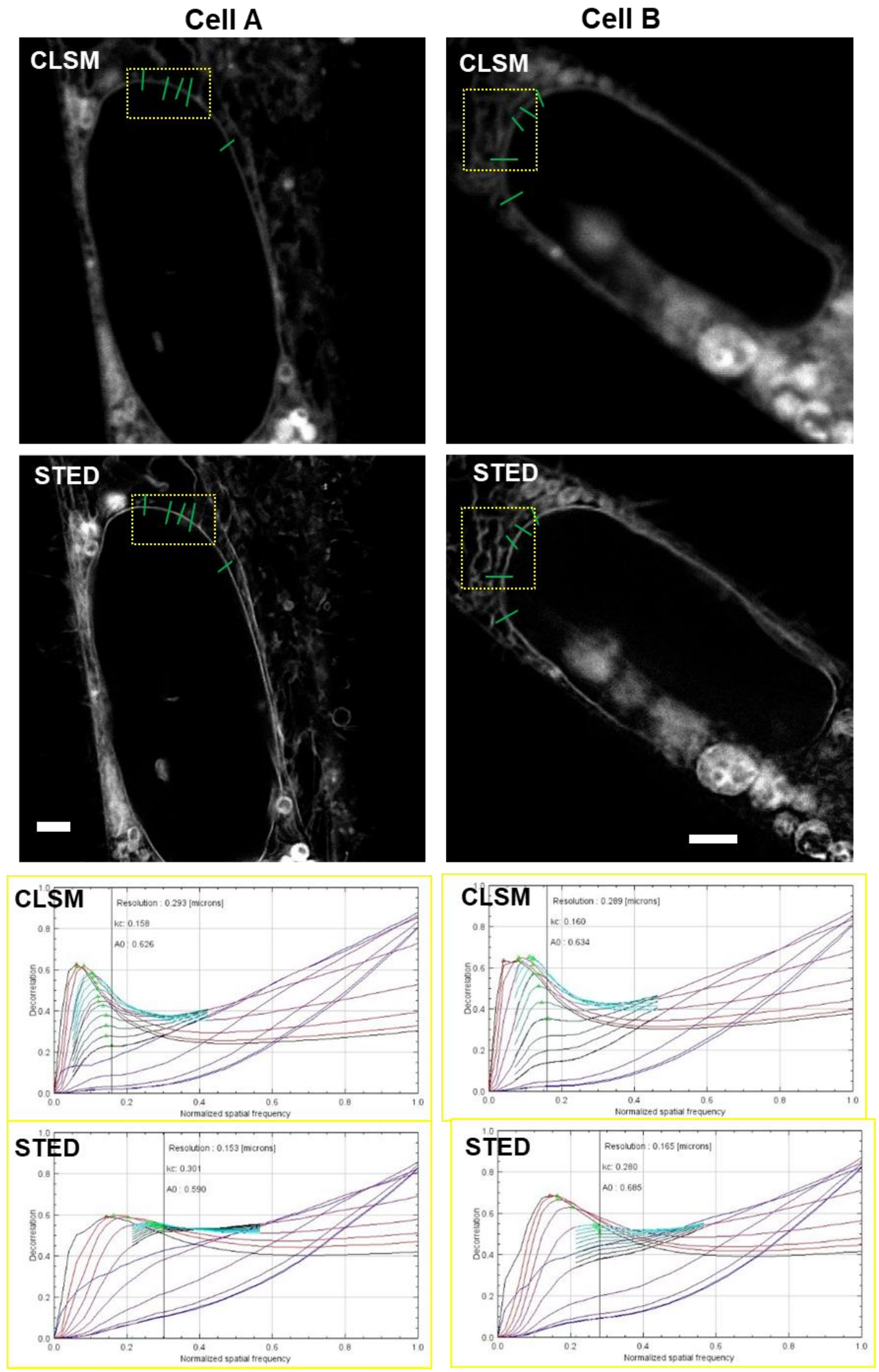

	Average NM ROI FWHM (nm)	Image resolution (yellow box) (nm)
Cell A CLSM	371	293
Cell A STED	154	153
Cell B CLSM	389	289
Cell B STED	186	165

Figure S7. CLSM and STED live MDA-MB 231 cell imaging of **1** (5 µM, 3 h incubation) used for resolution improvement analysis. Green lines used to measure NM ROI FWHM with average of five calculated. ROI in yellow box used for ImageJ image decorrelation analysis for image resolution, scale bars 2 µm.

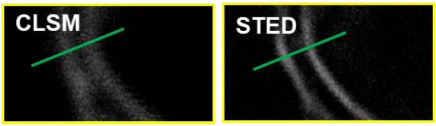

Figure S8. CLSM and STED live MDA-MB 231 cell imaging of **1** (5 μM, 3 h incubation) which was used for analysis in figure 6.

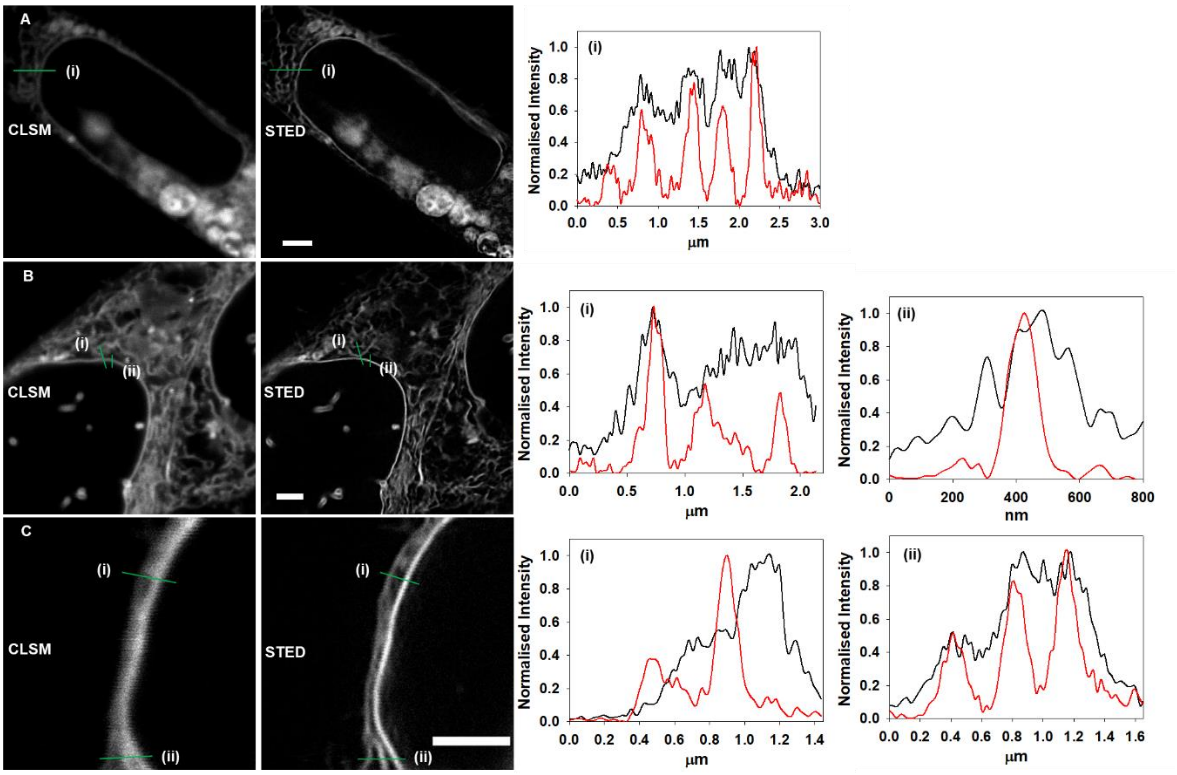

Figure S9. CLSM and STED imaging of live MDA-MB 231 cells with **1** (5 μM, 3 h incubation), intensity plots of corresponding green lines showing increased spatial resolution analysis of STED (red) compared with CLSM (black), scale bars 2 µm.

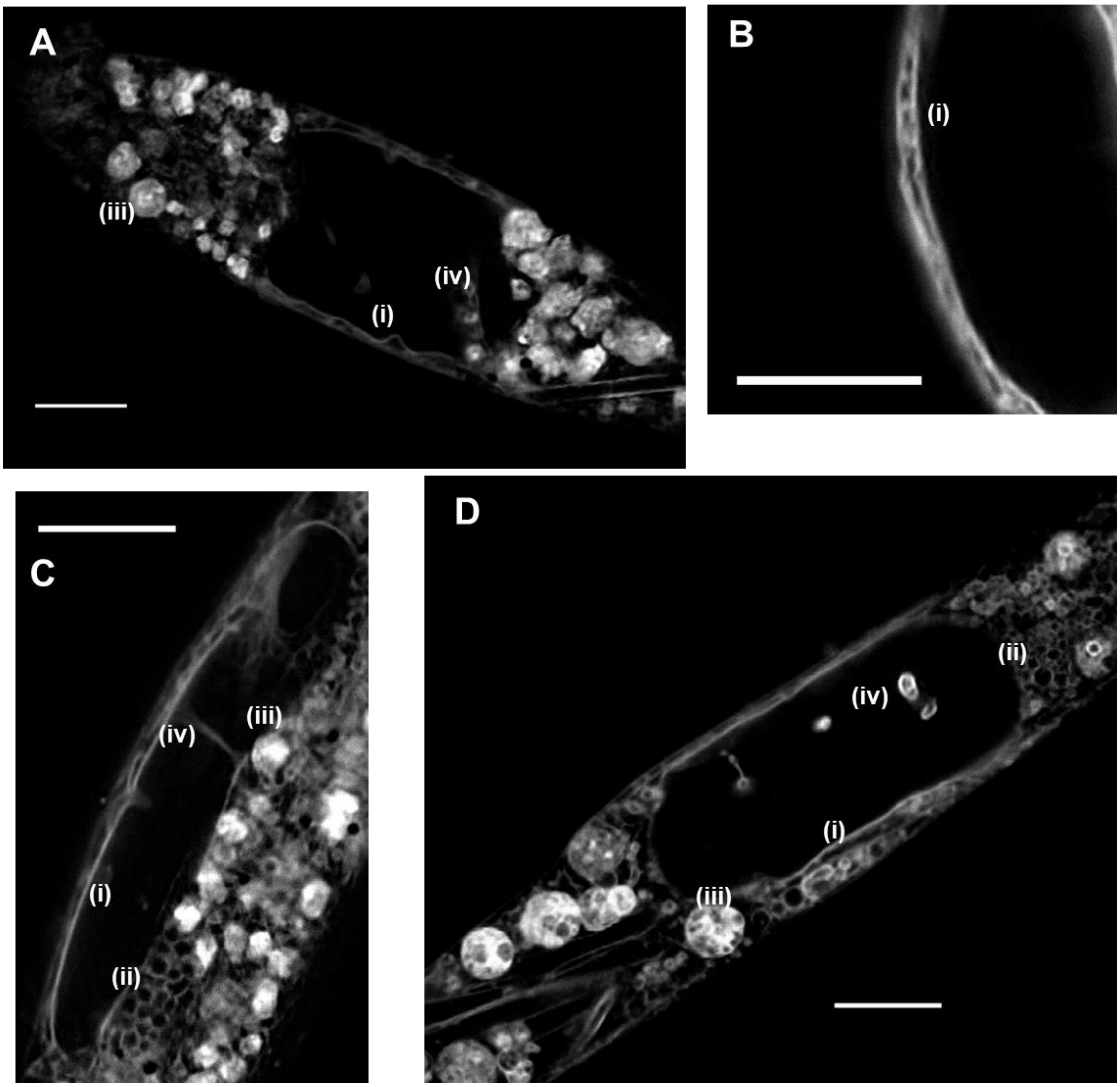

Figure S10. Live STED images of one plane of a z-stack of MDA-MB 231 cells incubated with **1** (5 µM, 3h incubation) showing (i) NM (ii) ER (iii) LV and (iv) NI, scale bar 5 µm, for z-stack movies see (A) z-stack range 0.6 µm, 7 steps, accumulation time 25 s, SI Movie S4, (B) z-stack range 0.5 µm, 6 steps, accumulation time 12 s, SI Movie S6, (C) z-stack range 0.6 µm, 6 steps, accumulation time 60 s, SI Movie S7, (D) z-stack range 7.5 µm, 26 steps, accumulation time 97 s, SI Movie S2. For additional z-stack movies see SI Movie S3 and S8A-B.

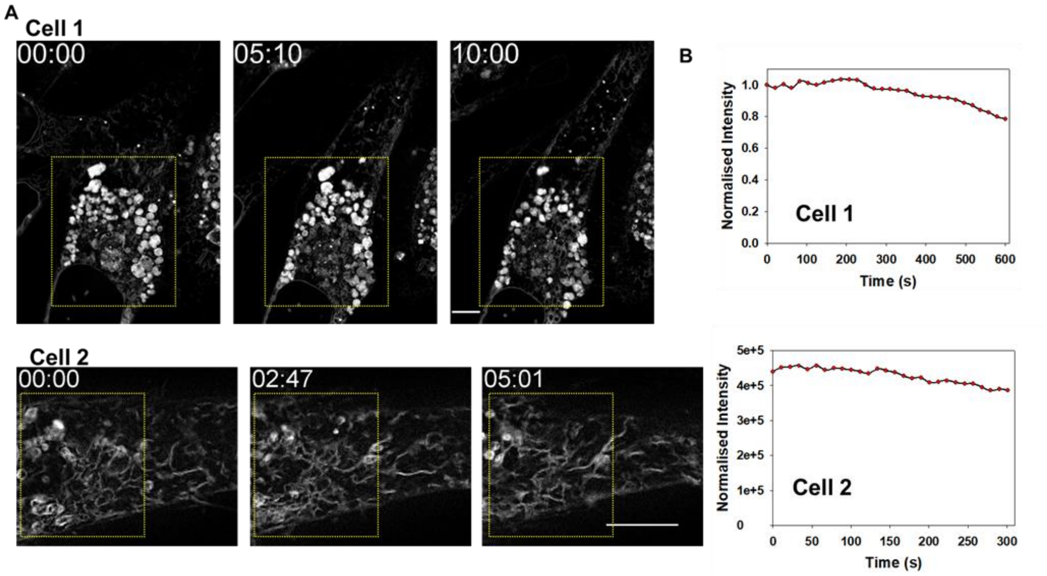

Figure S11. Continuous live MDA-MB 231 cell STED imaging following treatment with **1** (5 μM, 3h incubation) using DL at 30% power. (A) Cell 1, STED timelapse at 0, 5 and 10 min (STED image acquired every 20 sec) and cell 2 STED timelapse at 0, 2.5 and 5 min (STED image acquired every 11 sec), scale bars 5 μm. For full timelapse movies see SI Movie S1A-B). (B) Normalised fluorescence intensity of ROI (yellow box) over time.

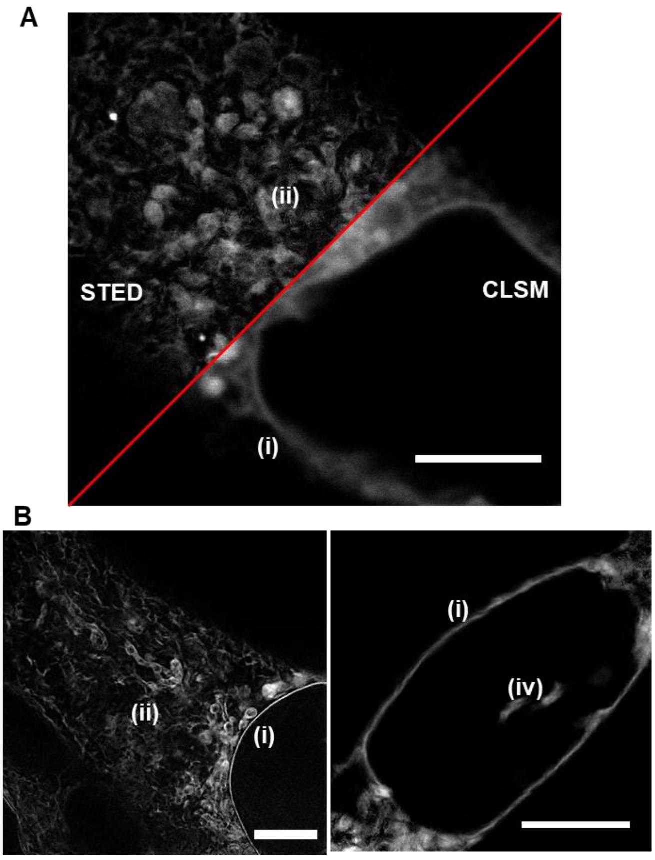

Figure S12. (A) Comparative CLSM and STED imaging of **2** (5 µM, 3 h incubation) in MDA-MB 231 cells. (B) Live cell STED imaging of **2** (5 µM, 3 h incubation). Identifiable subcellular features labelled as (i) NM, (ii) ER, (iii) LV (iv) NI, scale bars 5 µm.

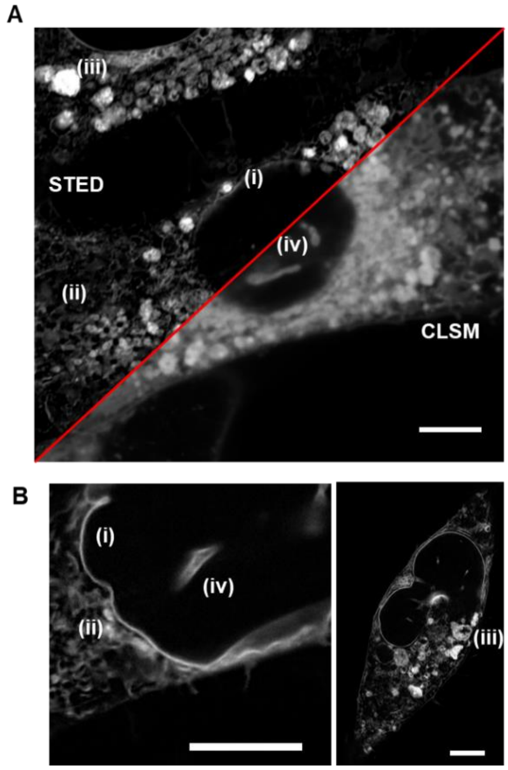

Figure S13. (A) Comparative CLSM and STED imaging of **3** (5 µM, 3 h incubation) in MDA-MB 231 cells. (B) Live cell STED imaging of **3** (5 µM, 3 h incubation). Identifiable subcellular features labelled as (i) NM, (ii) ER, (iii) LV (iv) NI, scale bars 5 µm.

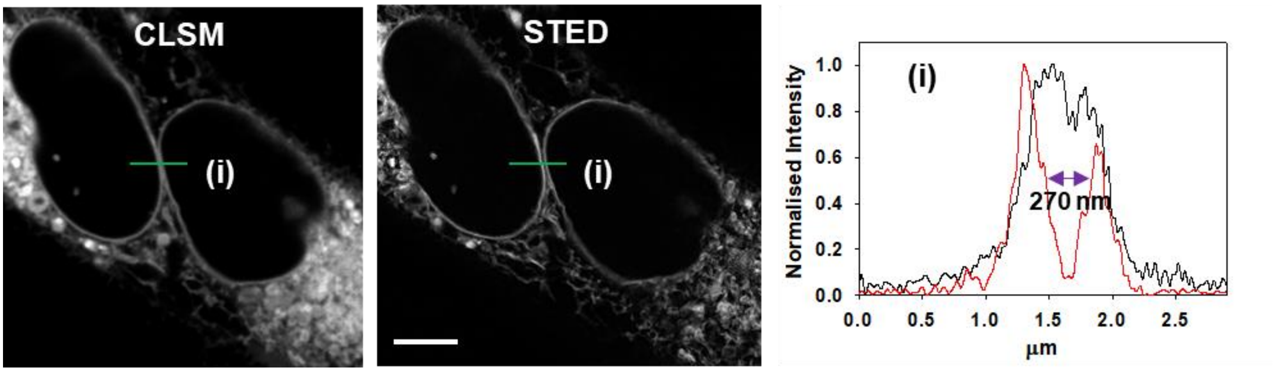

Figure S14. CLSM and STED imaging of **2** (5 µM, 3 h incubation) in MDA-MB 231 cells with intensity plot of corresponding green line showing increased spatial resolution analysis of STED (red) compared with CLSM (black), scale bar 5 µm.

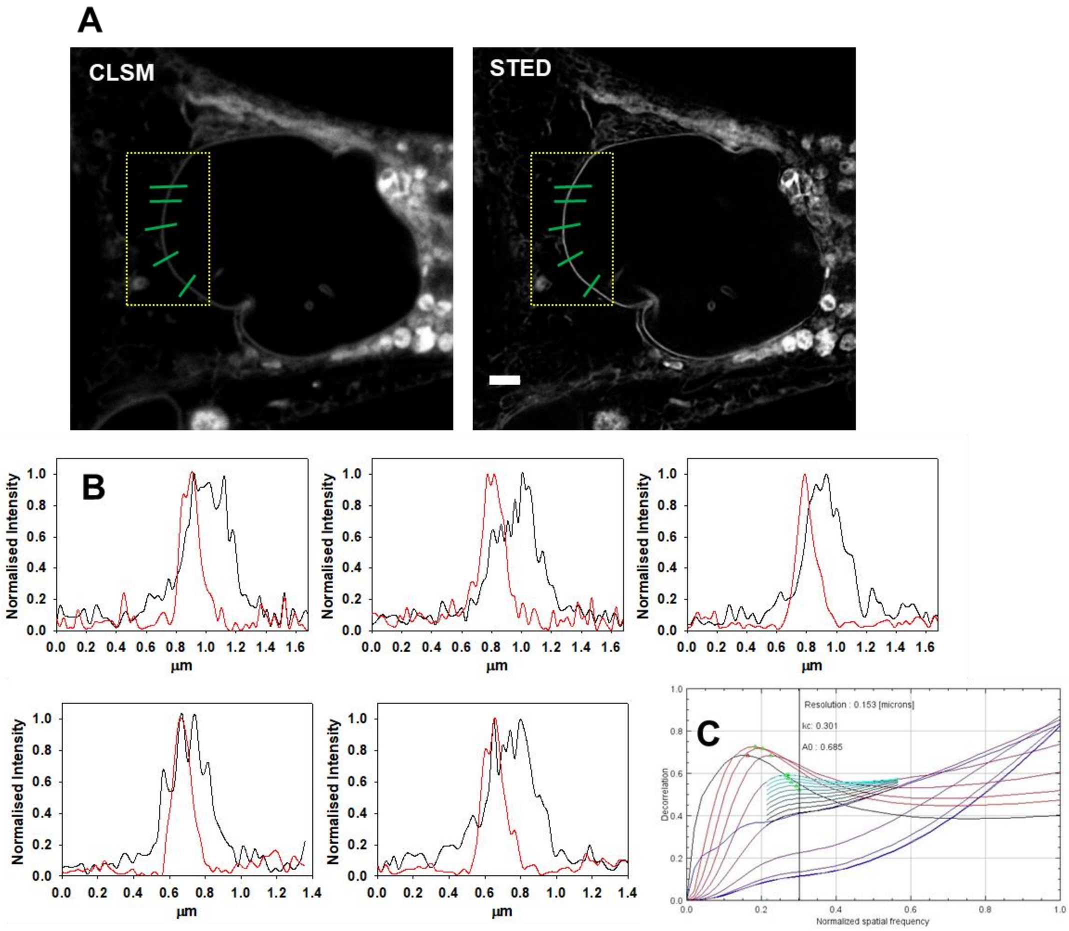

Figure S15. (A) CLSM and STED images of **1** (5 µM, 3 h incubation) in MDA-MB 231 cells showing NM ROI used for further analysis, scale bar 2 µm. (B) Intensity plots of green lines used to measure FWHM of ROIs along NM, STED (red) and CLSM (black), (C) ImageJ image decorrelation analysis measured for yellow box NM ROI.

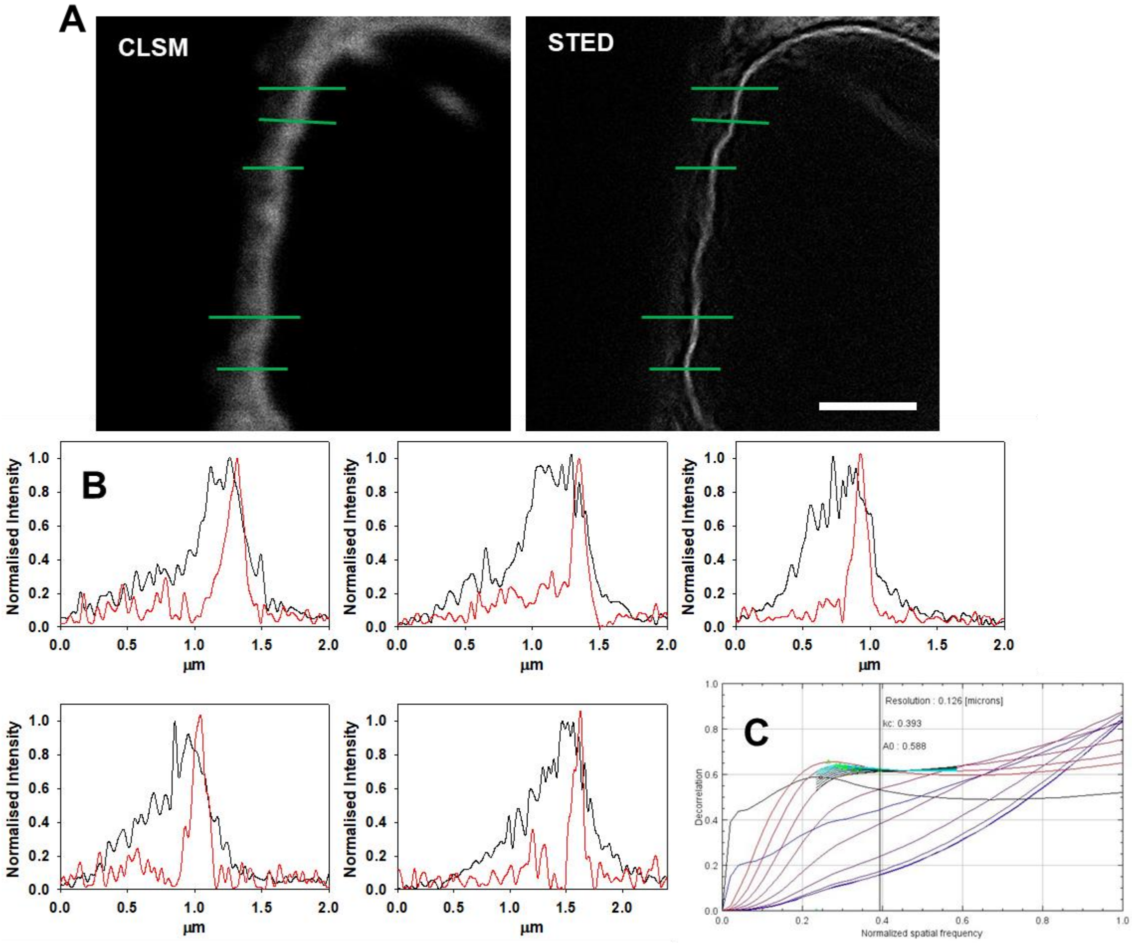

Figure S16. (A) CLSM and STED images of **2** (5 µM, 3 h incubation) in MDA-MB 231 cells showing NM region used for further analysis, scale bar 2 µm. (B) Intensity plots of green lines used to measure FWHM of ROIs along NM, STED (red) and CLSM (black), (C) ImageJ image decorrelation analysis measured for full image.

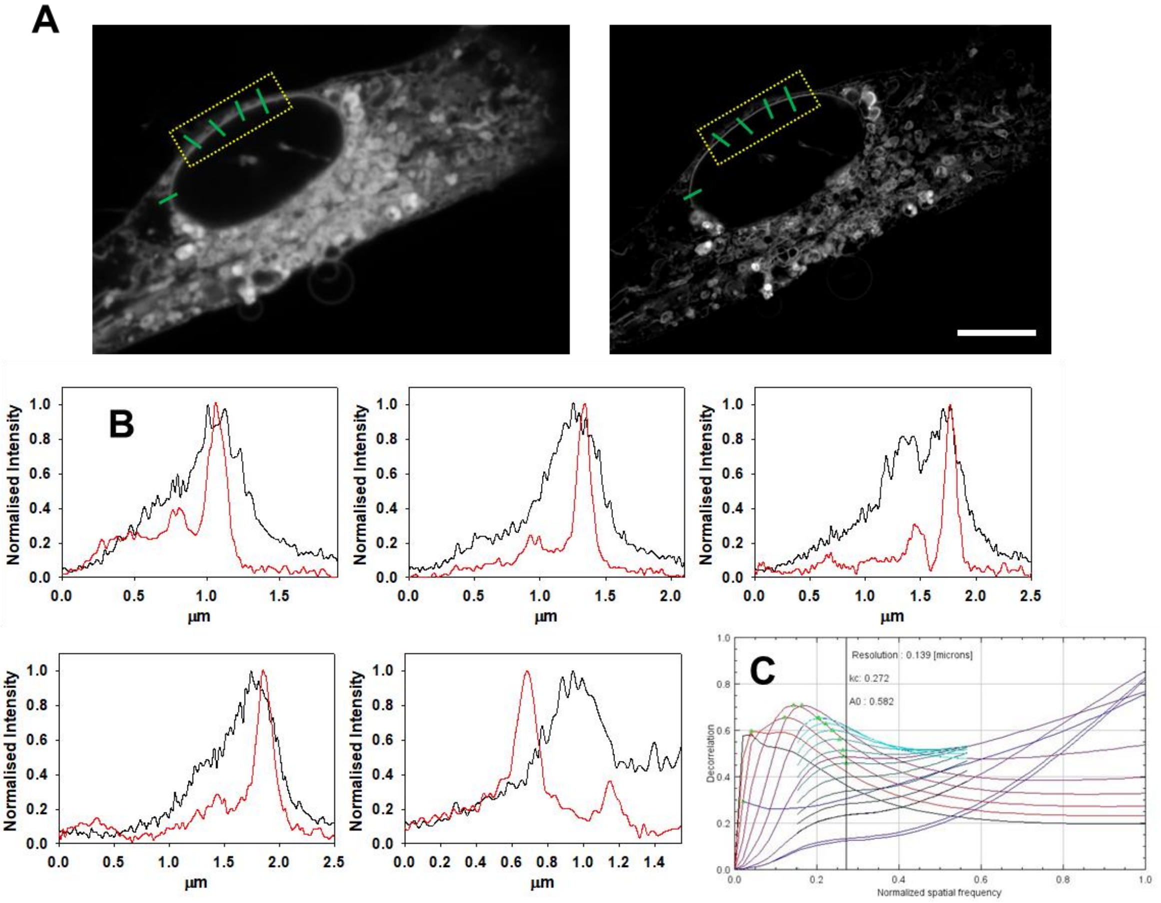

Figure S17. (A) CLSM and STED images of **3** (5 µM, 3 h incubation) in MDA-MB 231 cells showing NM region used for further analysis, scale bar 5 µm. (B) Intensity plots of green lines used to measure FWHM of ROIs along NM, STED (red) and CLSM (black), (C) ImageJ image decorrelation analysis measured for yellow box NM ROI.

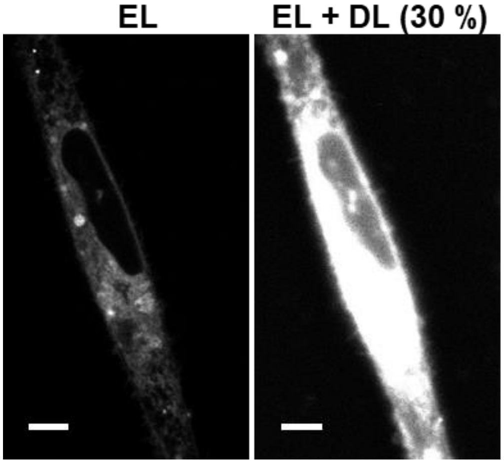

Figure S18. Live MDA-MB 231 cell imaging of **4** (5 µM, 3 h incubation) using EL (0.7 %) and EL (0.7 %) + DL (30 %), scale bar 5 µm .

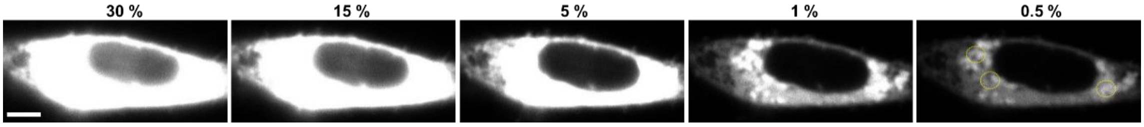

Figure S19. Imaging of **4** (5 µM, 3 h incubation) in live MDA-MB 231 cells using DL (30 – 0.5 %) showing saturation of image by DL, yellow circles showing cytosol ROIs used for average max intensity calculations, scale bar 5 µm, SI Movie S9.

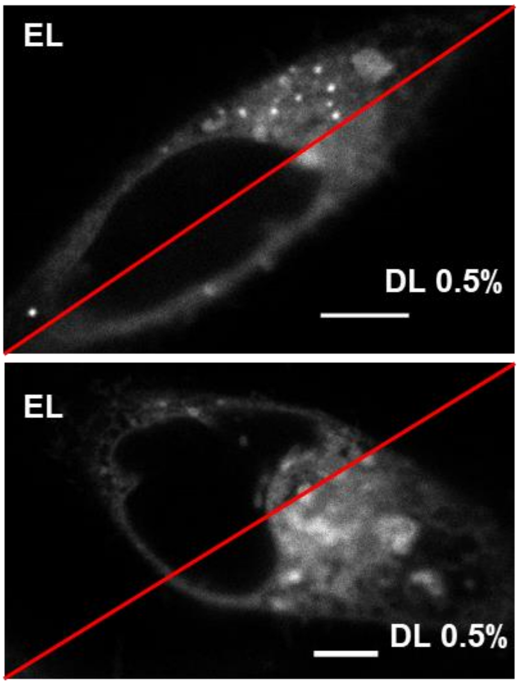

Figure S20. Repeat experiments imaging of **4** (5 µM, 3 h incubation) in live MDA-MB 231 cells using EL (CLSM) and DL alone (0.5 %) showing emission from same intra-cellular regions, scale bar 5 µm.

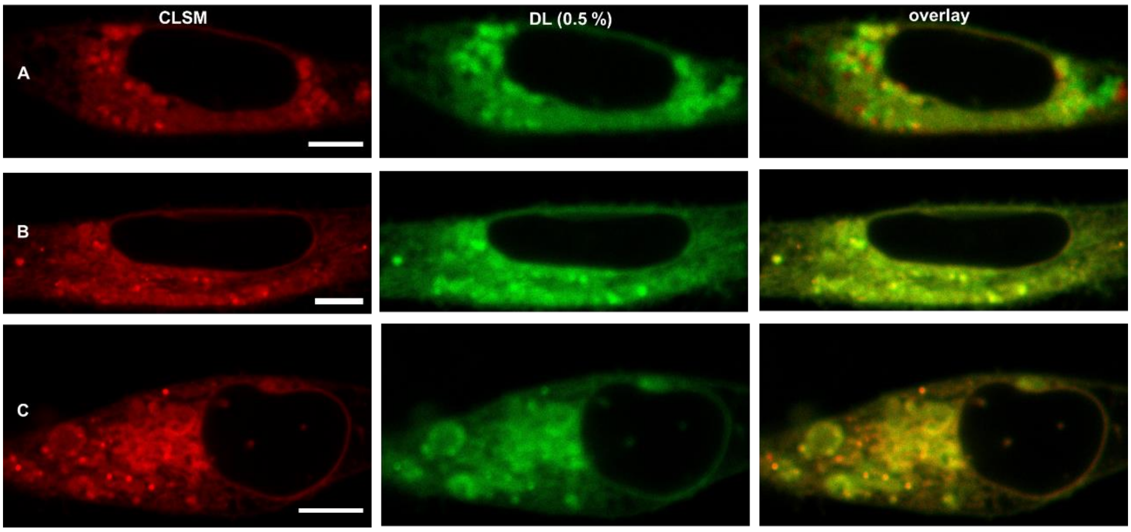

Figure S21. Live MDA-MB 231 cell imaging of **4** (5 µM, 3 h incubation) in cells A-C by EL (CLSM, red), DL alone (0.5%, green), and overlay in yellow, scale bar 5 µm.
